# Evaluation of the efficacy and safety of herbal medicine for treating work-related chronic low back pain: A study protocol for a multicenter, randomized, controlled, clinical trial

**DOI:** 10.1097/MD.0000000000016466

**Published:** 2019-07-26

**Authors:** Youme Ko, Bo-Hyoung Jang, Min-Seok Oh, Sun Joong Kim, Yun-Yeop Cha, Eun Jung Lee, Yun-Kyung Song, Seung-Gyu Ko

**Affiliations:** a Department of Science in Korean Medicine, Graduate School, Kyung Hee University; b Department of Korean Rehabilitation Medicine, College of Korean Medicine, Dae-Jeon University, Daejeon; c Department of Korean Rehabilitation Medicine, College of Korean Medicine, Semyung University, Jecheon; d Department of Korean Rehabilitation Medicine, College of Korean Medicine, Sang-ji University, Wonju; e Department of Korean Rehabilitation Medicine, College of Korean Medicine, Gachon University, Seongnam-si, The Republic of Korea.

**Keywords:** herbal medicine, korean medicine, sogyeonghwalhyeol-tang, work related chronic low back pain

## Abstract

**Introduction:**

In Korea, low back pain (LBP) which is occupation-related symptom is one of the major health issues owing to rapid industrialization. Even traditional Korean medicine has the long history in pain treatment, there still has been lack of supporting evidence on herbal prescription itself. Sogyeonghwalhyeol-tang, a Korean herbal medicine prescription, has been suggested as a medication for treating chronic LBP as well as work-related pains.

**Objective:**

This study aims to evaluate the clinical efficacy and safety of herbal medicine, Sogyeonghwalhyeol-tang on work-related chronic LBP patients.

**Method:**

This trial is designed as a multicenter, randomized, controlled, clinical trial. Seventy-two participants who have chief complaint of LBP in Korean medicine rehabilitation center will be randomly assigned to ether Sogyeonghwalhyeol-tang group or placebo group with a ratio of 1:1. They will receive assigned drugs in 4 weeks and follow-up for 2 weeks.

**Discussion:**

The result of this study will provide the valuable information for efficacy and safety of Sogyeonghwalhyeol-tang for patients with work-related chronic LBP.

## Abstract 2

Supplemental Digital Content is available in the text

## 1 Introduction

Work-related chronic low back pain (Wr-cLBP) is an exceedingly common health problem in industrialized countries. It is caused by repetitive posture, maintaining a fixed position for prolonged duration, and rapid work pace; the resulting disability can become a socioeconomic problem and lower an individuals’ quality of life. According to the 2018 annual report by the Korea Occupational Safety and Health Agency, work-related injuries or resulting disability of low back comprise 40.5% (3281/8105) of the overall work-related diseases or injuries.^[1]^

Wr-cLBP generally causes the loss of productivity, leading to economic burden, and needs long-term treatment care plan. Treatment with conventional medicine promotes health and prevents the disease to reduce and stabilize an individual's quality of their life. Conventional medical approaches generally include the use of non-pharmacologic treatments, such as physical therapy and chiropractic care, based on clinical practice guideline.^[2,3]^ However, traditional Korean medicine (TKM) utilizes multiple, individualized treatment methods, such as acupuncture, moxibustion, Chuna, and various herbal medicines to treat the overall discomfort because of Wr-cLBP with a holistic approach.^[4]^ As per the 2017 report by the Korean Medical Use and Consumption Survey, low back pain (LBP) is a frequent reason for seeking medical care at Korean medical institution, with a lifetime utilization of 52.7%.^[5]^ This result indicates that a considerable number of patients with LBP approach TKM institutions to treat their discomfort in Korea.

Sogyeonghwalhyeol-tang (SGHH) is addressed in one of the famous traditional Chinese medicine classics, Wanbinghuichun, and is presumed to mediate its effect by activating the blood and dredging the meridians and collaterals to treat abnormal musculoskeletal symptoms, such as pain, palsy, and stiffness.^[6]^ Several studies have reported on pain related to arthritis and neurologic symptoms.^[7–10]^

Although TKM has a long history of treating Wr-cLBP, no well-designed clinical trial has been undertaken to evaluate the potential efficacy of SGHH in the Korean population. Therefore, we will examine the feasibility of a full, randomized, placebo-controlled clinical trial to evaluate the efficacy of SGHH on Korean patients with work-related LBP to provide more conclusive evidence of its therapeutic efficacy.

### 1.1 Study setting

This pilot trial will be a multicenter, double-blind, randomized, parallel-group, placebo-controlled clinical trial that will be conducted in 4 university-affiliated TKM hospitals: Gil oriental medical hospital of Gachon university, Semyung University Second Affiliated oriental medical hospital at Jecheon, Dunsan Korean medicine hospital of Daejeon university, and oriental Medicine hospital of Sangji University.

**Figure 1:**
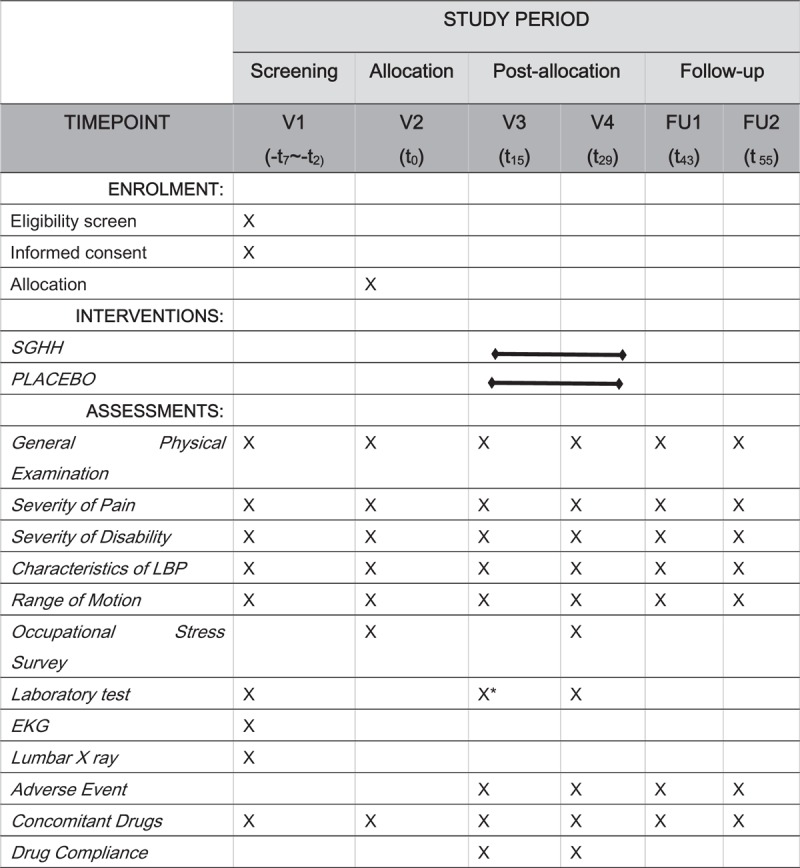
Study flow of the SGHH clinical trial. SGHH = Sogyeonghwalhyeol-tang.

On confirming their participation in this trial, the recruited participants will undergo an evaluation for identifying eligible candidates. A total of 72 eligible participants will have a 7-day run-in period before randomization. After randomization, the participants will receive treatment for 4weeks, with additional 4 weeks of follow-up, during which they will visit the clinic once every 2 weeks. The flow chart describing the trial protocol is shown in Figure 1.

### 1.2 Eligibility criteria

A total of 72 patients with symptoms of work-related LBP will be enrolled from among the susceptible working population. Considering the participants’ occupation, we reviewed the frequency of patients with Wr-cLBP in each of the participating institutions. Thereafter, we constituted an advisory committee comprising TKM rehabilitation experts and the principal investigators from each site for finalizing the target population. Consequently, we obtained information on the occupation of the study population, which are as follows: blue collar workers,^[11]^ hospital workers,^[12]^ office worker,^[13]^ and transit workers^[14]^ (Table 1).

**Table 1:**
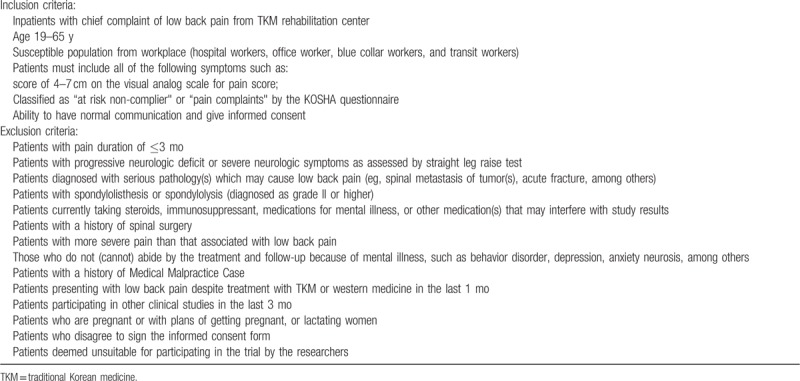
Eligibility criteria.

### 1.3 Interventions

After randomization, the participants will be started on 9 g of SGHH or placebo per day, for 4 weeks. Both groups will consume the given drugs orally, with warm water, 3 times a day between meals. The investigational drug, SGHH, is a well-known herbal formulation in traditional medicine for treating various types of pains caused by arthritis, sciatica, gout, and back pain.^[15]^

In this trial, granulated extract of SGHH (manufactured by Hanpoong Pharm and Foods Co., Ltd.) will be used for convenience. Each dose packet contains 0.83 g of Paeoniae radix, 0.66 g of Angelicae radix, 0.66 g of Rehmaniae radix, 0.66 g of Atractylodis japonica Koidzumi, 0.66 g of Cnidii rhizome, 0.66 g of Persicae semen, 0.66 g of Poria, 0.50 g of Achyranthis radix, 0.50 g of Clematidis radix, 0.50 g of Sinomeni caulis et rhizome, 0.50 g of Notopterygii rhizome, 0.50 g of Saposhnikoviae radix, 0.50 g of Gentianae scabrae radix, 0.50 g of Aurantii nobilis pericarpium, 0.33 g of Angelicae dahuricae radix, 0.33 g of Glycyrrhizae radix, and 0.33 g of Zingiberis rhizome. Moreover, the placebo will be manufactured relatively similar in appearance to SGHH, under strict quality-control system such that the participants cannot distinguish between the 2. Placebo contains 5.1 g of lactose, 3.0 g of cornstarch, and 0.3 g of Ssanghwa herbal flavor.

To improve adherence of investigational drug, we will ask participants to return the unused drugs for drug compliance at every study visit.

### 1.4 Outcomes

To evaluate the intensity of the LBP, we have selected 3 assessment tools such as: assessing pain intensity by numeric rating scale (NRS),^[16]^ functional status by the Korean version of the Roland Morris Disability Questionnaire (RMDQ),^[17]^ and quality of life by European Quality of life 5 Dimension (EQ5D).^[18,19]^ All questionnaires will be completed by the participants on their own at each follow-up visit.

### 1.5 Primary outcome

#### 1.5.1 NRS

The 10-point numerical scale is used to assess the intensity of LBP on a scale of 0 (no pain) to 10 (worst pain). The participants will be asked to mark their pain intensity on the scale at each visit. The change in NRS score for pain intensity between the 2 groups will be analyzed.

### 1.6 Secondary outcome

#### 1.6.1 Roland morris disability questionnaire

The Korean version of RMDQ, the most responsive questionnaire that reflects the gradual clinical changes, will be used to evaluate the disability of daily life in this study. It consists of 24 items representing general physical functions that are likely to be affected by LBP. Higher numbers on the questionnaire's scale reflect greater levels of disability.

#### 1.6.2 European Quality of life 5 dimension

The Korean version of EQ-5D is a validated measure for assessing the health-related quality of life and consists of 2 types of measures—the descriptive system of EQ-5D and VAS of EQ (EQ-VAS). The descriptive system comprises 5 dimensions, including mobility, self-care, usual activities, pain/discomfort, and anxiety/depression, and each dimension includes 3 levels of severity of discomfort to daily life. The EQ-VAS is used as a quantitative measurement of health status self-reported by the participants.

#### 1.6.3 Safety assessment

Safety assessment, including physical examination, complete blood count test, blood biochemistry, renal function test (blood urea nitrogen [BUN], and creatinine), and liver function test (alanine transaminase [ALT], aspartate transaminase [AST], alkaline phosphatase [ALP], and gamma-glutamyl transferase [γ-GT]) will be conducted during screening visit and at visit number 4.^[20]^ BUN, creatinine, ALT, AST, and γ-GT are additionally assessed at visit number 3, and moreover, the vital signs will be checked at every visit.

### 1.7 Sample size

This trial is a pilot study to examine the feasibility of a full randomized controlled trial for evaluating the efficacy of SGHH and to determine the effect size for further large-scale studies. A sample cohort comprising 72 patients has been considered after assessing patients’ frequency of clinic visits, availability of study staff, amount of funding, and dropout rates at each participating site.

### 1.8 Recruitment

Participant recruitment will take place in 4 university affiliated Korean medicine hospitals. Each site may include participants by means of website, hospital bulletin board, and local community-based newspaper publishing recruitment posters.

### 1.9 Randomization and allocation

The eligible participant will be randomly assigned to either the treatment (SGHH) or placebo group with an allocation ratio of 1:1. The random assignment will be done by the web-based service provided by the contract research organization (CRO), Institute of Safety and Effectiveness Evaluation for Korean Medicine (ISEE). The random sequence will be generated by an independent study staff from ISEE using the SAS 6.1 program, and stratified by the participating institutions using block sizes of 2 and 4.

Once the participant is randomly assigned to a group, either the participant or the investigator will be made aware of the type of intervention the participant will receive until the end of the study period. The interventional agents will be packaged similarly such that they are indistinguishable by appearance, and distributed by independent study site-assigned pharmacists or assistants who will be blinded to the random allocation. In case of withdrawal symptoms or occurrence of urgent medical conditions, the site's principal investigator will directly inform the CRO and sponsor to unblind the participant's intervention according to the standard operating procedures (SOPs).

### 1.10 Statistical management

Data analysis will be conducted by both intent-to-treat (ITT) and per-protocol (PP) analysis by an independent statistics expert, blinded to the study. Independent sample *t* test for continuous data and *χ*^2^ or Fisher exact tests for categorical data will be used. The repeated measures analysis of variance will be used to compare the baseline measurements with visit number 4, and follow-up numbers 1 and 2. Comparison of means of the continuous variables (ie, NRS, RMDQ and EQ5D scores) will be expressed as mean differences with 95% confidence intervals (CIs). Missing data will be adjusted using the last-observation carried-forward imputation method. Statistical analyses will be performed using SPSS 23.0 program and *P* < .05 will be considered statistically significant. There will be no interim analysis unless the pattern identification decide to stop the trial and access authority to the final dataset will be only given to the pattern identification.

### 1.11 Data monitoring

The monitoring of collected data and research performances will be conducted by ISEE on a regular basis. The monitoring will begin when the first enrolled participant completes the required number of visits. All the participating sites will be monitored based on the SOPs during the trial progress. Auditing is not scheduled for this pilot study. Data validation and verification, such as double data entry and range checks for data values, will be performed for ensuring data quality. Any other committee, such as a coordinating center, a steering committee, an endpoint adjudication committee, among others, will not be applicable in the present study. No auditing will be performed as well.

### 1.12 Harms

All participants will be informed of potential adverse events (AEs) occurring during the trial and cases of AE will be reported to the site trial staff immediately. Each AE will be recorded in the electronic medical record and case report form by the site trial staff and all AEs will be assessed for causality. If serious adverse events (SAEs) occur, the site staff will report to the site principle investigator and institutional review board (IRB) at the earliest. After receiving an SAE report, the site principle investigator will decide on the continuation of the participant in the trial.

### 1.13 Withdrawal and dropout

Participants may withdraw from the trial at any time, without penalty. If violation of eligibility criteria or situations, such as concomitant drugs, low drug compliance occurs, principle investigator decide the termination of the study. All the progress notes on withdrawal and dropouts are recorded on the CRFs with reasons.

### 1.14 Ethics and dissemination

This study has been designed in accordance with the amended Declaration of Helsinki and the regulations of the “Good Clinical Practice” principles of the Korea Food and Drug Administration. It has been approved by the IRB of the 4 participating hospitals (Gil oriental medical hospital of Gachon university, 17–101; Semyung University Second Affiliated oriental medical hospital at Jecheon, 2016-04-01; Dunsan Korean medicine hospital of Daejeon university, DJDSKH-16-BM-02, and oriental Medicine hospital of Sangji University, SJIRB-DRUG-16-001). The current version of the protocol is 1.1 and it has been formulated according to the Standard Protocol Items: Recommendations for Interventional Trials (SPIRIT) Statement (see Supplementary Appendix 3). All items from the World Health Organization Trial Registration Data Set have been drawn. All participants will receive detailed explanation and sufficient time to determine trial participation, and hand-written informed consent will be obtained from all participants before proceeding with any of the trial procedures. All personal information will be *stored* in locked cabinets in secured areas to protect confidentiality. When important protocol changes occur, the changes must be approved by the IRBs before implementation. The results of this study will be published in a scientific journal.

## 2 Discussion

Wr-cLBP is a common disease affecting the patients’ overall quality of life in industrial countries. In this study, we will explore the efficacy and safety of the herbal medicine extract, SGHH, in patients with work-related LBP. To our best knowledge, this is the first randomized controlled trial that will evaluate the efficacy of an herbal medicinal extract as a single intervention in cases of Wr-cLBP.

There are several limitations to this trial. First, because of the limited number of participating institutions and considering their possible enrollment conditions, we have restricted the type of occupations that will be targeted for participant enrollment. Second, owing to the limited research grant for the pilot study, our sample size is limited, and the study results cannot be generalized. However, attempts will be made to control or reduce any potential biases that may affect the trial results.

Nevertheless, this trial will provide valuable information, such as study design, outcome variables, pain characteristics of each occupation, and statistical power for sample size calculation for future large-scale trials, and the future development of general clinical guidelines for Wr-cLBP in the field of Korean medicine.

## Author contributions

**Conceptualization:** Min-Seok Oh, Sun Joong Kim, Yun-Yeop Cha, Eun Jung Lee, Yunkyung Song.

**Methodology:** Bo-Hyoung Jang, Min-Seok Oh, Sun Joong Kim, Yun-Yeop Cha, Yunkyung Song.

**Project administration:** Yunkyung Song, Seung-Gyu Ko.

**Supervision:** Yunkyung Song, Seung-Gyu Ko.

**Writing – original draft:** Youme Ko.

**Writing – review & editing:** Bo-Hyoung Jang, Min-Seok Oh, Sun Joong Kim, Yun-Yeop Cha, Yunkyung Song.

## Supplementary Material

Supplemental Digital Content
