# Supplementary material for: Evaluation of the efficacy and safety of herbal medicine for treating work-related chronic low back pain: A study protocol for a multicenter, randomized, controlled, clinical trial
Source: Medicine (Baltimore). 2019 Jul 26;98(30):e16466. doi: 10.1097/MD.0000000000016466 (PMC6708960; doi:10.1097/MD.0000000000016466)
Supplement: Supplemental Digital Content [file medi-98-e16466-s001.pdf]

[Appendix] Informed Consent Form (ver 1.1)

Study Title: Evaluation of the Efficacy and Safety of Herbal Medicine for Treating Work-related Chronic Low Back Pain: A Study Protocol for a multicenter, randomized, controlled, clinical trial

□ I have read the participant information sheet and I understood the purpose, methods, expected effect, possible risk, and information management collected in the study with a full explanation.

□ I have had the opportunity to ask questions about it and any questions that I have asked have been answered to my satisfaction.

□ I was also informed that I can withdraw the agreement and receive appropriate treatment if any adverse event occurs.

□ I understood the explanation about collecting, using and providing personal information.

□ I have a copy of this consent form and information sheet.

□ I have been given sufficient time to consider and I consent voluntarily to participate as a participant in this research.

Participant

Print Name __________________ Signature ___________________ Date ____________________

Legal representative (if necessary) (Relationship:)

Print Name __________________ Signature ___________________ Date ____________________

Witness (if necessary)

Print Name __________________ Signature ___________________ Date ____________________

Researcher/person taking the consent

Print Name __________________ Signature ___________________ Date ____________________
